# Lactate based caproate production with *Clostridium drakei* and process control of *Acetobacterium woodii* via lactate dependent *in situ* electrolysis

**DOI:** 10.3389/fbioe.2023.1212044

**Published:** 2023-06-23

**Authors:** Jan Herzog, Alexander Mook, Tyll Utesch, Frank R. Bengelsdorf, An-Ping Zeng

**Affiliations:** ^1^ Institute of Bioprocess and Biosystems Engineering, Hamburg University of Technology, Hamburg, Germany; ^2^ Institute of Molecular Biology and Biotechnology of Prokaryotes, Ulm University, Ulm, Germany; ^3^ Synthetic Biology and Bioengineering Lab, School of Science, Westlake University, Hangzhou, China

**Keywords:** *Acetobacterium woodii*, *Clostridium drakei*, caproate, lactate, bioelectrochemical system, *in situ* electrolysis, carbon fixation, process control

## Abstract

Syngas fermentation processes with acetogens represent a promising process for the reduction of CO_2_ emissions alongside bulk chemical production. However, to fully realize this potential the thermodynamic limits of acetogens need to be considered when designing a fermentation process. An adjustable supply of H_2_ as electron donor plays a key role in autotrophic product formation. In this study an anaerobic laboratory scale continuously stirred tank reactor was equipped with an All-in-One electrode allowing for *in-situ* H_2_ generation via electrolysis. Furthermore, this system was coupled to online lactate measurements to control the co-culture of a recombinant lactate-producing *Acetobacterium woodii* strain and a lactate-consuming *Clostridium drakei* strain to produce caproate. When *C. drakei* was grown in batch cultivations with lactate as substrate, 1.6 g·L^−1^ caproate were produced. Furthermore, lactate production of the *A. woodii* mutant strain could manually be stopped and reinitiated by controlling the electrolysis. Applying this automated process control, lactate production of the *A. woodii* mutant strain could be halted to achieve a steady lactate concentration. In a co-culture experiment with the *A. woodii* mutant strain and the *C. drakei* strain, the automated process control was able to dynamically react to changing lactate concentrations and adjust H_2_ formation respectively. This study confirms the potential of *C. drakei* as medium chain fatty acid producer in a lactate-mediated, autotrophic co-cultivation with an engineered *A. woodii* strain. Moreover, the monitoring and control strategy presented in this study reinforces the case for autotrophically produced lactate as a transfer metabolite in defined co-cultivations for value-added chemical production.

## 1 Introduction

Fermentation processes with syngas have been proposed in the past as a promising technology to reduce the accumulation of carbon dioxide (CO_2_) in the atmosphere and thus prevent negative effects of rapid climate change (Latif et al., 2014; Bengelsdorf and Dürre, 2017). Among the many microorganisms which can grow using hydrogen (H_2_), carbon monoxide (CO) and CO_2_ via hydrogenesis, methanogenesis and acetogenesis (Dürre and Eikmanns, 2015), acetogens show a variety of promising industrial application possibilities (Abubackar et al., 2011; Liew et al., 2016; Liew et al., 2022). *Acetobacterium woodii* is one of these microorganisms which can use CO_2_ as sole carbon source, reducing it via the Wood-Ljungdahl pathway to C1 and C2 compounds such as formate, acetate or ethanol (Drake et al., 2008). *A. woodii* has been studied extensively in the past (Balch et al., 1977; Poehlein et al., 2012; Beck et al., 2020) and is considered to be a model acetogen for sodium bioenergetics (Biegel and Müller, 2010). The main natural product of *A. woodii* is acetate. It has been genetically accessible since 1994 (Strätz et al., 1994) and recombinant strains have been constructed for the production of C3 compounds like acetone (Hoffmeister et al., 2016) and lactate (Mook et al., 2022). Recently, lactate has drawn special attention for being a promising feed compound to produce higher-value products via chain elongation (Detman et al., 2019; Liu et al., 2020). Caproic acid is an important chemical which can be produced via chain elongation from lactate (Kucek et al., 2016a; Zhu et al., 2017). It has a wide array of applications in the food industry as precursor for aromas (Desbois, 2012), in the cosmetic and pharmaceutical industry and even as possible precursor for biofuels (Cavalcante et al., 2017). Given that the production of caproic acid is currently mainly fossil-based, its sustainable production through fermentation of waste products is gaining increased interest (Wasewar and Shende, 2011). Among the bacteria which can produce caproate are *Clostridium kluyveri, Clostridium carboxidivorans,* and *Clostridium drakei* (Wirth and Dürre, 2021), the latter which can also grow on lactate as primary carbon source (Küsel et al., 2000). The production of caproate in *C. drakei* happens via reverse ß-oxidation. The *bcd/hcs* gene cluster and its respective enzymes catalyze the elongation of acetyl-CoA to butyryl-CoA and subsequently to hexanoyl-CoA from which caproate is then derived. *C. drakei* has already been successfully proven to produce caproate from lactate in a co-cultivation with *A. woodii* (Herzog et al., 2022) and showed great potential for caproate production from CO_2_.

While syngas fermentation processes could contribute to the reduction of CO_2_ by reusing this waste component as primary carbon-source for the production of industrial chemicals, they also require H_2_ as reduction equivalent (Wood, 1991). H_2_ demand added up to 94 Mt worldwide in 2021 and is still almost entirely produced from fossil sources, mainly coal and natural gas. The associated CO_2_ emissions of H_2_ production added up to 900 Mt in 2021. H_2_ will be a critical element in the energy transition and an important technology towards a climate friendly industry. It is estimated that in 2030 only 24 Mt could be produced from low-emission sources (IEA, 2022). This implies that for the near future the great majority of H_2_ will be produced from fossil sources. To ensure a true low-emission syngas fermentation, H_2_ would have to either be produced from gasification of biomass (Abubackar et al., 2011) or via electrolysis powered by renewable energy. A promising technology to produce H_2_ directly in the bioreactor is the All-in-One (AiO) electrode (Utesch and Zeng, 2018). This highly flexible electrode can be inserted in any standard bioreactor to apply *in situ* electrolysis during the running fermentation (Utesch and Zeng, 2018). This AiO-electrode has been successfully used for the control and optimization of electricity-aided microbial production of 1,3-propanediol and lipids (Utesch et al., 2019; Arbter et al., 2022). Powering this electrode with electricity from renewable sources could also turn the syngas fermentation into a true low-emission process.

The aim of this work was the characterization of *C. drakei* growing on lactate as carbon source in a stirred-tank bioreactor and proving the feasibility of a lactate based-process control. First, we identified the lactate consumption and caproate production rates of *C. drakei* grown on lactate. Furthermore, we designed and programmed a lactate-based process control which can be applied in any lactate producing fermentation. Applying this to the co-culture process of *C. drakei* and *A. woodii* (Herzog et al., 2022) the lactate production rate of *A. woodii* can be adjusted to the maximum lactate consumption rate of *C. drakei* determined through a dynamic operation of the AiO-electrode. This control should minimize H_2_ production to the required minimum and prevent an excess waste of energy and H_2_. In times of high energy prices, minimizing energy costs plays a major role in economic feasibility of a new process.

## 2 Materials and methods

### 2.1 Microorganism and medium

Construction of *A. woodii* [P_*bgaL*_ *_ldhD*_NFP] is described in Mook et al. (2022) while the *C. drakei* wild-type strain was obtained from the German Collection of Microorganisms (DSMZ 12750). Cultivation medium for both strains was prepared according to Herzog et al. (2022). For *C. drakei* cultivations, 10 g·L^−1^ DL-lactate was added as carbon source to the medium. Pre-cultures of *A. woodii* [P_*bgaL*_ *_ldhD_*NFP] were cultivated heterotrophically using fructose (2 g·L^−1^) at 30°C in non-agitated anaerobic serum bottles. *C. drakei* pre-cultures were cultivated under the same conditions with the exception of using a 75:25 ratio (v/v) of lactate and fructose. The pre-cultures were incubated for 30–33 h until reaching an optical density at 600 nm (OD_600_) of 3.3 ± 0.4 for *C. drakei* and 2.0 ± 0.9 for *A. woodii* [P_*bgaL*_ *_ldhD_*NFP].

### 2.2 Stirred-tank reactor fermentations and analytics

Batch fermentations were carried out in a 2.0 L stirred-tank bioreactor (KSF 2000, Bioengineering AG, Wald, Switzerland) with a working volume of 1.4 L. The medium was sterilized *in situ* at 121°C for 20 min before each fermentation. Then, the cultivation medium was degassed with N_2_ to ensure anaerobic conditions. An oxygen reduction potential (ORP) sensor was installed to ensure an ORP value lower than −280 mV prior to inoculation. After degassing, the reactor was sparged constantly with CO_2_ at a gas flow rate of 0.9 L·h^−1^ with an open tube L-sparger. *A. woodii* [P_*bgaL*_ *_ldhD_*NFP] cultures were agitated with three Rushton disk turbines (d = 40 mm) at a stirrer speed of 800 rpm (P V^−1^ = 2.4 W·L^−1^), while *C. drakei* culture were agitated at 210 rpm (P V^−1^ = 0.04 W·L^−1^). The temperature was controlled at 30°C and pH was measured via a pH sensor and controlled at pH 7.0 ± 0.2 by the addition of a 5 M KOH solution. H_2_ for the fermentations with *A. woodii* [P_*bgaL*_ *_ldhD_*NFP] was supplied by the AiO-electrode via *in situ* electrolysis. The rod-shaped electrode consists of a platinized titan mesh as working electrode (platin coating thickness d = 2.5 µm, coating density ρ = 50 g·m^-2^) on the outside where H_2_ is produced and which is in contact with the fermentation medium. The working electrode is separated from the counter electrode by a ceramic separator, creating a counter electrode chamber with an exhaust duct at the top. This enables the produced O_2_ to leave the bioreactor without interfering with the cultivation medium. The working electrode surface is 75 cm^2^ while the counter electrode surface is 14 cm^2^ (Utesch and Zeng, 2018). A scheme of the AiO-electrode is shown in the Supplementary Figure S1. The AiO-electrode was operated without reference electrode to maintain a constant current of 600 mA (*j* = 8 mA·cm^−2^, E_cell_ = 4.2 ± 0.3 V) by a power supply (2231A-30-3; Keithley, Solon, OH, United States). *C. drakei* cultivations were conducted without the AiO-electrode. The OD_600_ of the pre-culture was used to calculate the necessary inoculation volume to start the fermentations to an OD_600_ of 0.15 for *A. woodii* [P_*bgaL*__*ldhD*_NFP] and 0.2 for *C. drakei* respectively. For *A. woodii* [P_*bgaL*__*ldhD*_NFP] cultivations, the production of lactate was induced with the addition of 0.3 g·L^−1^ lactose. Induction was conducted when the batch culture had reached an OD_600_ of 0.48 ± 0.13. The online lactate measurement was carried out with a TRACE C2 Control (TRACE Analytics GmbH, Braunschweig, Germany) using a dialysis probe with a membrane for low lactate concentrations. The automated current adjustment for H_2_ production by the AiO-electrode was regulated by a LabVIEW script containing a proportional-integral-derivate controller (PID) algorithm. The process variable lactate concentration (g·L^−1^) was transmitted with 4-20 mA via an analog digital converter (USB-6001; National Instruments Corp., Austin, TX, United States) to the host computer. The control variable was the electrical current (A) applied to the AiO-electrode by the power supply via RS232 communication. The set point was the desired lactate concentration. The derivative value of the controller was set to 0 while proportional gain was set to 0.01 and the integral time to 2 min with a sampling time of 10 ms. For co-cultivations, *A. woodii* [P_*bgaL*_ *_ldhD_*NFP] was inoculated first into the bioreactor and grown under the same conditions as in the pure culture experiments (T = 30°C; pH = 7.0; P V^−1^ = 2.4 W·L^−1^; F_CO2_ = 0.9 L·h^−1^; I_AiO_ = 600 mA; V_0_ = 1.4 L) until 0.4 g·L^−1^ of lactate were produced. The automated process control was programmed to switch off the AiO-electrode at this lactate concentration. Subsequently, 90 mL of a *C. drakei* pre-culture were added to the bioreactor. Cell growth was determined with OD_600_ measurements (photospectrometry), while lactate, acetate, butyrate and formate concentrations were determined via HPLC. The exhaust gas composition and flow were monitored constantly with a mass flow meter (EL-FLOW prestige, Bronkhorst High-Tech B.V., Ruurlo, Netherlands) and a mass spectrometer (Omnistar GDS 300, Pfeiffer Vacuum GmbH, Asslar, Germany). All analytical methods are described in detail in Herzog et al. (2022). The experimental plan of the fermentations conducted in this study is shown in Table 1.

**TABLE 1:** Experimental plan of all fermentations described in this study.

Strain	Substrate	pH [-]	T [°C]	V_0_ [L]	N_st_ [rpm]	F_CO2_ [L h^−1^]	I_AiO_ [mA]	t_AiO, off_ [h]	Control
*C. drakei*	Lactate	7.0	30	1.4	210	0.9	-	-	-
*A. woodii* [P_*bgaL*_ *_ldhD_*NFP]	H_2_/CO_2_	7.0	30	1.4	800	0.9	600	-	-
*A. woodii* [P_*bgaL*_ *_ldhD_*NFP]	H_2_/CO_2_	7.0	30	1.4	800	0.9	600	6	manually
*A. woodii* [P_*bgaL*_ *_ldhD_*NFP] + *C. drakei*	H_2_/CO_2_ (Lactate)	7.0	30	1.4	800	0.9	600	3	automated

## 3 Results

### 3.1 Cultivation of *C. drakei* on lactate as substrate

**FIGURE 1:**
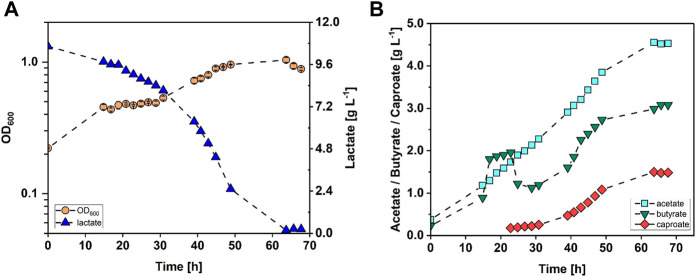
Stirred-tank batch cultivation of *C. drakei* with lactate as substrate. **(A)** Optical cell density (OD_600_, orange circles), lactate concentration measured in the medium (blue triangles); **(B)** acetate concentration (cyan squares), butyrate concentration (green triangles), caproate concentration measured in the medium (red diamonds). (T = 30°C; pH = 7.0; P V^−1^ = 0.04 W·L^−1^; F_CO2_ = 0.9 L·h^−1^; V_0_ = 1.4 L).

*C. drakei* was cultivated in a stirred tank reactor as described in Chapter 2.2 with lactate being the sole substrate. The cultivation was reproduced (*n* = 2) and average values with standard deviation are mentioned in this section. Representative data of only one fermentation are shown in Figure 1 (refer to Supplementary Figure S2 in the additional dataset). *C. drakei* cells grew with a growth rate of 0.04 h^−1^ in the first 15 h to an OD_600_ of 0.39 ± 0.12 and subsequently entered stationary phase. This phase lasted 16 h where the average OD_600_ added up to 0.41 ± 0.03. Afterwards, the *C. drakei* culture entered a second growth phase with µ = 0.04 h^−1^ which lasted for 20 h reaching an OD_600_ of 0.93 ± 0.08 after 49 h of fermentation time. Subsequently, the culture was stationary for the remaining 17 h of the process at a constant OD_600_ of 0.96 ± 0.04. Lactate concentrations in the medium started decreasing with the start of the fermentation. In the first 21 h, lactate was consumed at a rate of 0.06 g·L^−1^ h^−1^, reducing the initial concentration of 10.7 g·L^−1^ by 11% to 9.5 g·L^−1^. Then, at the end of the first stationary growth phase, the lactate consumption rate almost doubled to 0.11 g·L^−1^ h^−1^ and during the second exponential growth phase it reached a maximum value of 0.24 g·L^−1^ h^−1^. After 66 h of fermentation time, lactate concentration decreased to 0.13 ± 0.18 g·L^−1^. Acetate was produced throughout the fermentation at a constant rate of 0.06 g·L^−1^ h^−1^ and reached a final concentration of 4.53 ± 0.53 g·L^−1^. Butyrate was produced at a similar rate as acetate during the first 15 h. Afterwards, the production rate increased to 0.15 g·L^−1^ h^−1^, reaching a peak concentration of 1.57 ± 0.65 g·L^−1^ after 21 h of fermentation time. In the following 8 h, butyrate concentration decreased to 1.26 ± 0.27 g·L^−1^ due to reassimilation, before increasing again with a production rate of 0.10 g·L^−1^ h^−1^ until 47 h fermentation time. The concentration then increased further to a maximum of 3.14 ± 0.08 g·L^−1^ after 64 h with a reduced rate of 0.04 g·L^−1^ h^−1^. Caproate concentrations were detected in the medium after 23 h of fermentation. The caproate production rate added up to 0.01 g·L^−1^ h^−1^ in the first 31 h of fermentation time. Afterwards, the production rate increased to 0.06 g·L^−1^ h^−1^ between 39 h and an estimated 58 h of fermentation time. The maximum concentration of 1.56 g·L^−1^ was measured after 64 h when the culture had already become stationary.

### 3.2 *A. woodii* [P_*bgaL*_ *_ldhD_*NFP] gas fermentations with manually controlled lactate production

**FIGURE 2:**
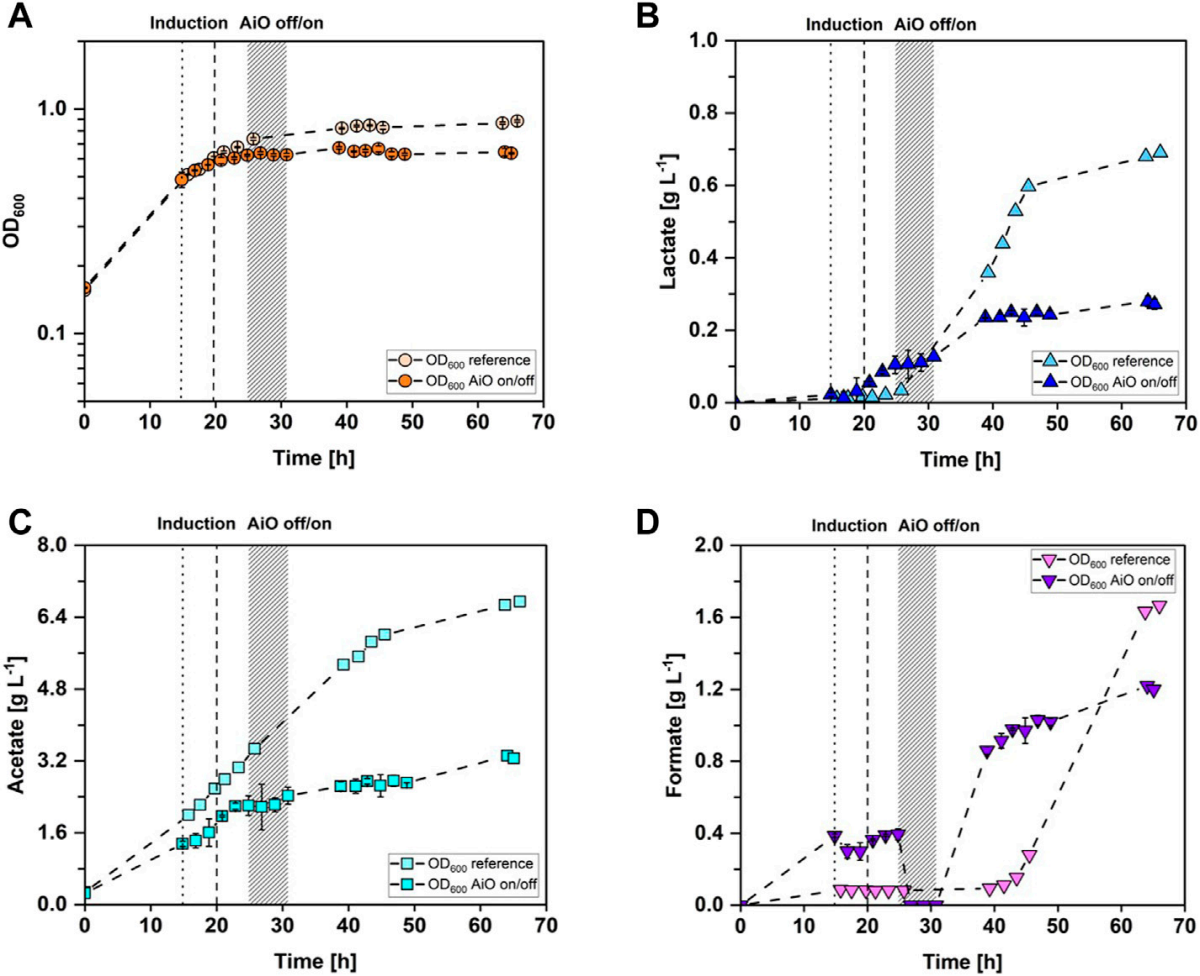
Stirred-tank batch cultivations of *A. woodii* [P_*bgaL*_ *_ldhD_*NFP] with manually controlled AiO-electrode as well as reference cultivation. **(A)** Optical cell density (OD_600_), reference cultivation in light orange circles and manual AiO-electrode control fermentation in darker orange circles. **(B)** lactate concentration measured in the medium, reference fermentation in light blue triangles, controlled fermentation in dark blue triangles; **(C)** acetate concentration; reference fermentation in light cyan squares and controlled fermentation in dark cyan squares; **(D)** formate concentration measured in the medium, reference fermentation in light purple triangles and controlled fermentation in dark purple triangles manual. The dotted line indicates the time point of induction of the manually controlled fermentation while the dashed line indicates the time point of induction of the reference fermentation. Both cultivations were induced with the addition of 0.3 g·L^−1^ lactose. The grey bar represents the time interval when the AiO-electrode was turned off and then on again. (T = 30°C; pH = 7.0; P V^−1^ = 2.4 W·L^−1^; F_CO2_ = 0.9 L·h^−1^; I_AiO_ = 600 mA; V_0_ = 1.4 L).

The concept of controlling the lactate production of *A. woodii* [P_*bgaL*_ *_ldhD_*NFP] by dynamic on-off switching of the AiO-electrode raises the question regarding the impact of a H_2_ limited phase on the lactate metabolism of *A. woodii* [P_*bgaL*_ *_ldhD_*NFP]. Specifically, it is unclear whether the lactate metabolism of *A. woodii* [P_*bgaL*_ *_ldhD_*NFP] would be halted permanently or if *A. woodii* [P_*bgaL*_ *_ldhD_*NFP] would resume lactate production when H_2_ is present in the medium again. To answer this question, a controlled batch fermentation with the AiO-electrode was conducted where 10 h after induction, the AiO-electrode was manually turned off for an interval of 6 h. It was then turned on again to see whether lactate production is resumed. For comparison, a reference batch fermentation with *A. woodii* [P_*bgaL*_ *_ldhD_*NFP] where the AiO-electrode was left on for the whole process time was also conducted (refer to Figure 2A). Both cultivations were inoculated from a pre-culture with an OD_600_ of 2.5 ± 0.3 and started growing with the maximum growth rate of 0.07 h^−1^. The reference fermentation reached the stationary phase after 26 h and stayed at an average OD_600_ of 0.85 ± 0.02 for the rest of the fermentation. The manually controlled cultivation entered stationary phase already after 25 h, at an OD_600_ of 0.62 ± 0.01 and stayed afterwards at an average OD_600_ of 0.64 ± 0.02 until the end of the fermentation. The course of the lactate concentration over time is shown in Figure 2B. The lactate production of the reference fermentation did not start until 6 h after induction. Then, lactate was produced constantly at a maximum rate of 0.05 g·h^−1^ until 25.5 h after induction, reaching a concentration of 0.60 g·L^−1^. Subsequently, the lactate production rate decreased to 0.01 g·h^−1^. The maximum lactate concentration of 0.69 g·L^−1^ was reached after 66 h of fermentation time. The lactate formation of the controlled fermentation began 2 h after induction with a rate of 0.02 g·h^−1^ and 8 h later, the production halted at a concentration of 0.10 g·L^−1^ due to switching off the AiO-electrode. The concentration stayed constant at an average of 0.11 ± 0.01 g·L^−1^ during the 6 h when no electrolysis was taking place. Afterwards, the lactate concentration increased at a rate of 0.02 g·h^−1^ for 8 h, reaching a concentration of 0.24 g·L^−1^. For the last 26 h of the controlled fermentation, the lactate concentration stayed constant at an average of 0.25 ± 0.02 g·L^−1^. The measured acetate concentration is shown in Figure 2C. The reference fermentation produced acetate constantly at a rate of 0.17 g·h^−1^ for 46 h. Then, the production rate decreased to 0.04 g·h^−1^ adding up to a final concentration of 6.8 g·L^−1^. The acetate concentration of the controlled fermentation increased in the beginning of the fermentation with a production rate of 0.11 g·h^−1^ until the AiO-electrode was switched off. Afterwards, the acetate concentration stayed constant at an average of 2.26 ± 0.11 g·L^−1^. In the last 26 h of the process, the acetate concentration increased slowly at a rate of 0.03 g·h^−1^, adding up to a final value of 3.26 g·L^−1^. The course of the formate concentration over time is depicted in Figure 2D. In the reference fermentation, almost no formate was accumulated in the first 42 h of the process (average 0.09 ± 0.01 g·L^−1^). Subsequently, formate concentration rapidly increased with a constant rate of 0.10 g·h^−1^ adding up to a final concentration of 1.67 g·L^−1^. The concentration of formate in the controlled cultivation increased at the beginning to an average of 0.51 ± 0.06 g·L^−1^ until the AiO-electrode was switched off. Then, all formate was consumed during the first 2 h without active electrolysis. Afterwards, formate accumulated at a rate of 0.15 g·h^−1^ during the 8 h after the AiO-electrode was reactivated. Subsequently, formate production slowed down, adding up to a final value of 1.58 g·L^−1^.

### 3.3 Automatically controlled lactate production in co-cultivation of *A. woodii* [P_*bgaL*_ *_ldhD_*NFP] and *C. drakei*

**FIGURE 3:**
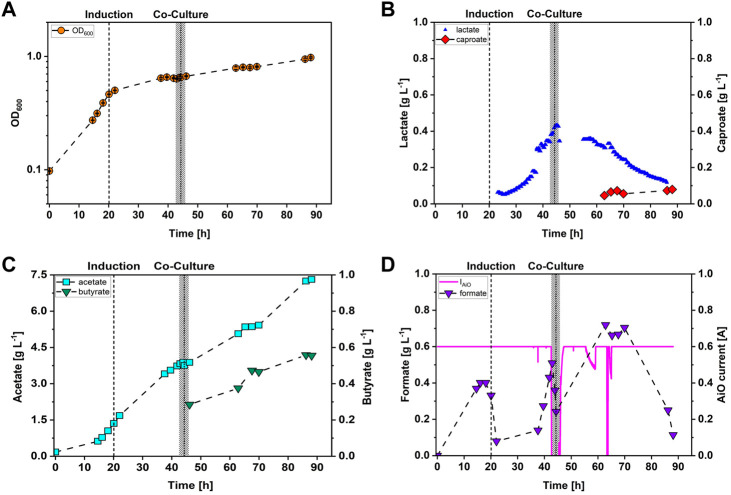
Stirred-tank batch co-cultivation of *A. woodii* [P_*bgaL*_ *_ldhD_*NFP] and *C. drakei* with automatically controlled AiO-electrode based on lactate concentration. **(A)** Optical cell density (OD_600_, orange circles); **(B)** lactate concentration measured in the medium with the TRACE C2 control (sample frequency 1 h, blue triangles), caproate concentrations measured in the medium (red diamonds); **(C)** acetate concentration measured in the medium (cyan squares), butyrate concentration measured in the medium (green triangles); **(D)** formate concentration measured in the medium (purple triangles), electrical current of the AiO-electrode (magenta line). The dashed line indicates the time point of induction of the culture with 0.3 g·L^−1^ lactose. The dotted line indicates the time point when *C. drakei* was added to the culture. The grey bar represents the time interval when the AiO-electrode was turned off automatically. (T = 30°C; pH = 7.0; P V^−1^ = 2.4 W·L^−1^; F_CO2_ = 0.9 L·h^−1^; I_AiO_ = 600 mA; V_0_ = 1.4 L; c_Lac, limit_ = 0.3 g·L^−1^).

A co-cultivation of *A. woodii* [P_*bgaL*_ *_ldhD_*NFP] and *C. drakei* was conducted to test the automated process control for feasibility. For this, the lactate threshold of the automation was set to 0.35 g·L^−1^ and *C. drakei* addition was timed 1.5 h after the process control had turned off the AiO-electrode. The threshold value was chosen to be lower than the average lactate concentration of 0.47 ± 0.11 g·L^−1^ based on previous data of fermentations with *A. woodii* [P_*bgaL*_ *_ldhD_*NFP] and the AiO-electrode. The results of the co-cultivation are shown in Figure 3. Cell growth of the *A. woodii* [P_*bgaL*_ *_ldhD_*NFP] strain increased after about 7 h with a maximum growth rate of 0.1 h^−1^. At an OD_600_ of 0.5 the cells were induced with 0.3 g·L^−1^ of lactose for lactate production and afterwards, the cell growth declined to 0.02 h^−1^. *C. drakei* was added to the bioreactor at an OD_600_ of 0.65, and afterwards, the combined OD_600_ of both strains kept increasing until reaching a final value of 1.0 after 88 h. After induction the *A. woodii* [P_*bgaL*_ *_ldhD_*NFP] strain started producing lactate with a maximum formation rate of 0.03 g·h^−1^. As seen in Figure 3B, the lactate measurement was recalibrated after 38 h due to deviation to a control measurement performed with HPLC. After 42.5 h, the measurement system detected lactate concentrations higher than the threshold and therefore, the AiO-electrode was automatically turned off. Due to residues of lactate in the pre-culture of *C. drakei*, the concentration increased slightly at the beginning of the co-cultivation phase. Furthermore, the lactate measurement system showed strong fluctuations subsequently which triggered the process control to turn on the AiO-electrode again, after 45.5 h and made a recalibration necessary. Subsequently, lactate concentrations decreased due to its consumption by *C. drakei* with a rate of 0.02 g·h^−1^. Caproate concentrations were first detected after 63 h and *C. drakei* kept producing caproate at a rate of 0.01 g·h^−1^. Final caproate concentrations added up to 0.08 g·L^−1^. Acetate was produced by the *A. woodii* [P_*bgaL*_ *_ldhD_*NFP] strain once it had entered the exponential growth phase reaching 3.8 g·L^−-1^ when *C. drakei* was added. Afterwards, both strains contributed to the increase of acetate throughout the subsequent fermentation. Final acetate concentrations added up to of 7.3 g·L^−1^. Butyrate was produced only during the co-cultivation phase by *C. drakei*, reaching 0.6 g·L^−1^ at the end of the fermentation. As shown in Figure 3D, formate was produced by the *A. woodii* [P_*bgaL*_ *_ldhD_*NFP] strain and concentrations reached a first peak after 16 h at 0.4 g·L^−1^. Subsequently, the *A. woodii* [P_*bgaL*_ *_ldhD_*NFP] strain consumed almost all of the formate before starting to produce again, reaching a second concentration peak at the moment the AiO-electrode was turned off at 0.5 g·L^−1^. During the period where no H_2_ was produced, the *A. woodii* [P_*bgaL*_ *_ldhD_*NFP] strain consumed parts of the formate and afterwards started producing it a third time, reaching a maximum concentration of 0.7 g·L^−1^. During the last 20 h of the process, formate was consumed again. The electrical current of the AiO-electrode was maintained constant at 600 mA until the electrode was turned off by the automation. During the co-cultivation of both strains, O_2_ started accumulating inside the counter electrode channel, causing an increase in electrical resistance and thus also in electrical voltage. Therefore, the electrical current decreased twice for short intervals, but refilling the counter electrode channel with culture medium resolved the issue. Relevant process performance parameters are listed in Table 2.

**TABLE 2:** Maximum cell density and growth rate, maximum lactate and caproate concentration as well as lactate and caproate formation rates of all fermentations described in this study.

Cultivation	OD_max_ [-]	µ_max_ [h^−1^]	Lac_max_ [g L^−1^]	q_lac, max_ [g h^-1^]	Cap_max_ [g L^−1^]	q_cap, max_ [g h^−1^]
*A. woodii* reference	0.88	0.07	0.69	0.05	-	-
*A. woodii* AiO on/off	0.67	0.07	0.28	0.02	-	-
*A. woodii* + *C. drakei* process control	0.98	0.10	0.39	0.03	0.08	0.01
*C. drakei*	0.99	0.04	10.67	−0.24	1.56	0.09

## 4 Discussion

*C. drakei* has been known to grow on lactate for some time (Küsel et al., 2000; Liou et al., 2005), yet to the authors best knowledge, this is the first study to show data of a stirred tank batch fermentation with *C. drakei* monoculture on lactate as carbon and energy source. Cell growth showed two growth phases (see Figure 1A), which could suggest the usage of a second substrate by *C. drakei* during the first growth phase, i.e., components of the yeast extract, given that during this phase only 7% (g g^−1^) of the lactate was consumed while cell density had already increased 1.8-fold. The maximum lactate consumption rate added up to 7.3 ± 1.7 g d^−1^ which is 20-fold higher than reported for previous co-cultivation (Herzog et al., 2022). The data also shows that the main caproate production takes place in the second growth phase, with only 17% of the total caproate being produced in the first 31 h of the fermentation and the remaining 83% in the following 35 h. It is assumed that the reduction of butyrate after 21 h of fermentation time is due to the reassimilation of butyrate to butyryl-CoA, possibly using ethanol as electron donor. Butyryl-CoA could have then be used in the chain elongation towards additional caproate. The caproate yield from lactate added up to 13% (g g^−1^) which is 2.3-fold lower than reported by the CPB6 strain of the *Oscillospiraceae* family in a similar process (Zhu et al., 2017), however these data were obtained with different bacteria and in a fed-batch fermentation. Data from other caproate producing novel *clostridia* species closely related to *Clostridium jeddahense* JCD in single cultures show comparable 18% (g g^−1^) yield from lactate (Liu et al., 2020). The process was conducted as batch fermentation, though as bottle fermentation and not as fermentation in a bioreactor as the presented results in this study. The volumetric productivity of 0.6 g·L^−1^ d^−1^ for caproate is 10-fold higher than reported recently by us in a co-cultivation (Herzog et al., 2022), suggesting that the potential for caproate production in the co-cultivation set up is not yet exhausted. Changing the fermentation mode from batch to fed-batch or continuous mode would help increase the volumetric productivity (Kucek et al., 2016a). Furthermore, the possibility to increase the chain-elongation rate of acetate to butyrate and then to caproate should be investigated, given that caproate adds up to only 23% of the three main products, while acetate accounts for almost double (43%). Testing feeding strategies and operating the process with an *in situ* product recovery technique might improve overall caproate yield (Kucek et al., 2016a; Kucek et al., 2016b).

To determine if the lactate production of *A. woodii* [P_*bgaL*_ *_ldhD_*NFP] is controllable, the AiO-electrode was switched off for an interval of 6 h during the controlled batch fermentation with *A. woodii* [P_*bgaL*_ *_ldhD_*NFP] (refer to Chapter 3.2). As Figure 2B shows, lactate production was successfully halted for 6 h and resumed after the AiO-electrode was switched on again. Lactate concentration increased 2.1-fold after the halting period at the same formation rate of 0.02 g·h^−1^ as before, which indicates that a lactate control by adjusting H_2_ production as proposed is possible. The lactate production afterwards only lasted for 8 h before entering a stationary phase, however a similar trend can be seen in the reference fermentation, where the lactate formation rate was reduced 5-fold at about the same time, 30 h after induction. A comparable effect was also observed in the recently published *A. woodii* [P_*bgaL*_ *_ldhD_*NFP] fermentation with the AiO-electrode, where lactate production stops 26 h after induction (Herzog et al., 2022). The 6 h of H_2_ limitation influenced cell growth and acetate production as well. Cell density differed on average 23% ± 4% from the reference fermentation while acetate added up to a 52% ± 2% lower concentration in comparison to the reference after the H_2_ limitation phase. However, the fermentation where the AiO-electrode was temporarily switched off showed lower product rates, as well as an earlier stationary growth phase already before the switching off of the electrode. Acetate formation rate was 1.3-fold and lactate formation rate 2.6-fold lower than compared to the reference fermentation. A possible explanation, next to slight differences in induction, could be, the inherent energy-limited regime for this autotrophic fermentation process. One indicator for energy-limitation in acetogens is the formation of formate, as the formyl-THF synthetase, catalyzing the conversion of formate to formyl-THF is ATP dependent (Moon et al., 2021). Disruption of Na^+^ homeostasis, and thereby ATP-synthase activity, has led to increased formate production in *Thermoanaerobacter kivui* (Yang and Drake, 1989) and *A. woodii* (Schwarz et al., 2022). The *A. woodii* [P_*bgaL*_ *_ldhD_*NFP] culture where the AiO-electrode was regulated accumulated 19 times more formate before the H_2_ limitation phase than the reference fermentation (see Figure 2D), hinting at a bottleneck in C1 and energy metabolism, even before the electrode was turned off. Interestingly, in the AiO-electrode off phase with no available H_2,_ the accumulated formate was quickly consumed. Oxidation of formate yields CO_2_ and H_2_ which probably allow for basic cell maintenance and even some product formation (Moon et al., 2021) as seen with the slightly increased acetate concentration. As H_2_-supply is reestablished, growth and production of acetate, lactate and formate is immediately resumed with formate production rates of up to 0.15 g·h^−1^. The reference fermentation neared a comparable formation rate of 0.10 g·h^−1^ 11 h later than the process with manual AiO-electrode control. As the reference fermentation constantly produced lactate, it stands to reason that the ATP-negative production of lactate (Bertsch and Müller, 2015; Mook et al., 2022) in the low H_2_ process with the AiO-electrode (Herzog et al., 2022) leads to energetic bottlenecks over the course of the fermentation. This in turn results in formate accumulation, which is even more severe and starts earlier when the electrode is turned off mid-fermentation.

In this study we proposed a controlled lactate production in *A. woodii* [P_*bgaL*_ *_ldhD_*NFP] to ideally match the lactate production rate to the maximum lactate consumption rate of *C. drakei* in a co-culture process. This would decrease H_2_ production to the necessary minimum and would reduce energy costs greatly. As the data in Figure 3 show, the controlled lactate production was successfully implemented. Once the lactate online measurement detected the defined threshold, the process control turned the AiO-electrode automatically off. As the lactate concentration decreased, the AiO-electrode was turned on again, showing that the process control can react dynamically to changing lactate levels. However, a reliable working online measurement system is necessary for exact automation. Frequent reference measurements and automated calibration cycles should provide more steady data stream. The comparison of the lactate production and consumption rates of *A. woodii* [P_*bgaL*_ *_ldhD_*NFP] (0.03 g·h^−1^) and *C. drakei* (0.24 g·h^−1^) determined in this study indicates that *C. drakei* has a higher rate of lactate consumption than *A. woodii* [P_*bgaL*_ *_ldhD_*NFP] has in production. It therefore seems that controlling lactate production is not necessary given that *C. drakei* could consume lactate faster than *A. woodii* [P_*bgaL*_ *_ldhD_*NFP] produces. However, the process with AiO-electrode as described here does not allow H_2_ production rates higher than 10 mmol·L^−1^·h^−1^ at the moment, which results in a H_2_ limitation of *A. woodii* [P_*bgaL*_ *_ldhD_*NFP] and thus lower lactate production rates. The H_2_ limitation and the resulting low lactate concentration also caused substrate limitation for *C. drakei*, resulting in lower caproate concentrations. As reported recently, cultivating *A. woodii* [P_*bgaL*_ *_ldhD_*NFP] with a H_2_ surplus of 971 mmol·L^−1^·h^−1^, the maximum lactate production rate can reach up to 0.21 g·h^−1^ (Herzog et al., 2022), which demonstrates that more efficient *A. woodii* [P_*bgaL*_ *_ldhD_*NFP] processes would benefit from the lactate process control presented in this study. Furthermore, this maximum lactate uptake rate of *C. drakei* was only reached in the last third of the fermentation (Figure 1A). In the first 15 h of the process, the lactate uptake rate was 4.9-fold lower. More importantly, in the co-cultivation where the lactate control is supposed to be implemented, lactate uptake rates of *C. drakei* reached only 0.01 g·h^−1^ and did not increase although the co-cultivation phase lasted 45 h. This indicates, that during a co-cultivation of *C. drakei* and *A. woodii* [P_*bgaL*_ *_ldhD_*NFP] with these low lactate concentrations, the lactate process control could reduce the amount of H_2_ being produced via electrolysis without limiting the availability of lactate for *C. drakei*, if lactate is already present in the medium.

## Data Availability

The datasets presented in this study can be found in online repositories. The names of the repository/repositories and accession number(s) can be found below: Mendeley data, doi: 10.17632/gmjvy88nd5.1 https://data.mendeley.com/datasets/gmjvy88nd5.
