# Supplementary material for: Lactate based caproate production with Clostridium drakei and process control of Acetobacterium woodii via lactate dependent in situ electrolysis
Source: Front Bioeng Biotechnol. 2023 Jun 23;11:1212044. doi: 10.3389/fbioe.2023.1212044 (PMC10327822; doi:10.3389/fbioe.2023.1212044)
Supplement: Supplementary file 1 [file DataSheet1.docx]

Supplementary Material

**
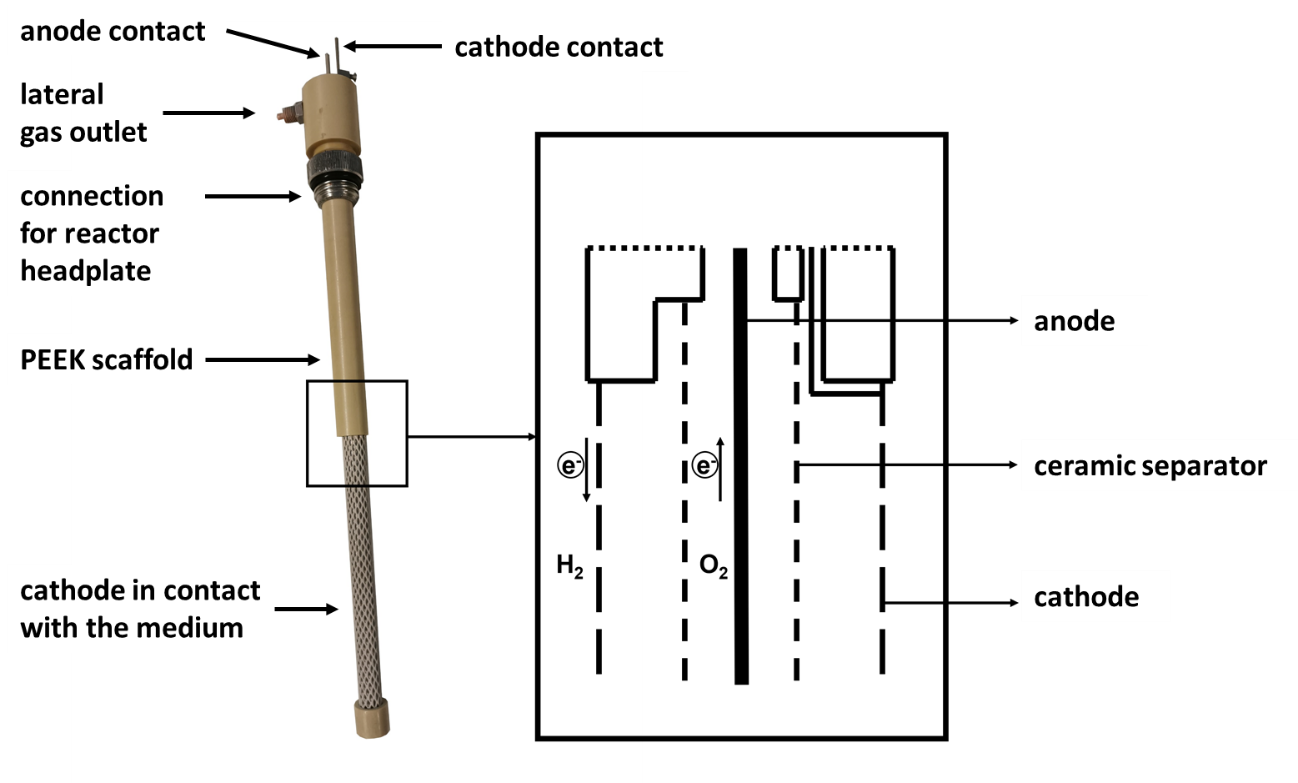
**

**Figure S1:** Simplified scheme of the All-in-One electrode for in-situ electrolysis (Herzog et al. 2022. Eng. Life Sci. 23, e2100169. doi:10.1002/elsc.202100169)


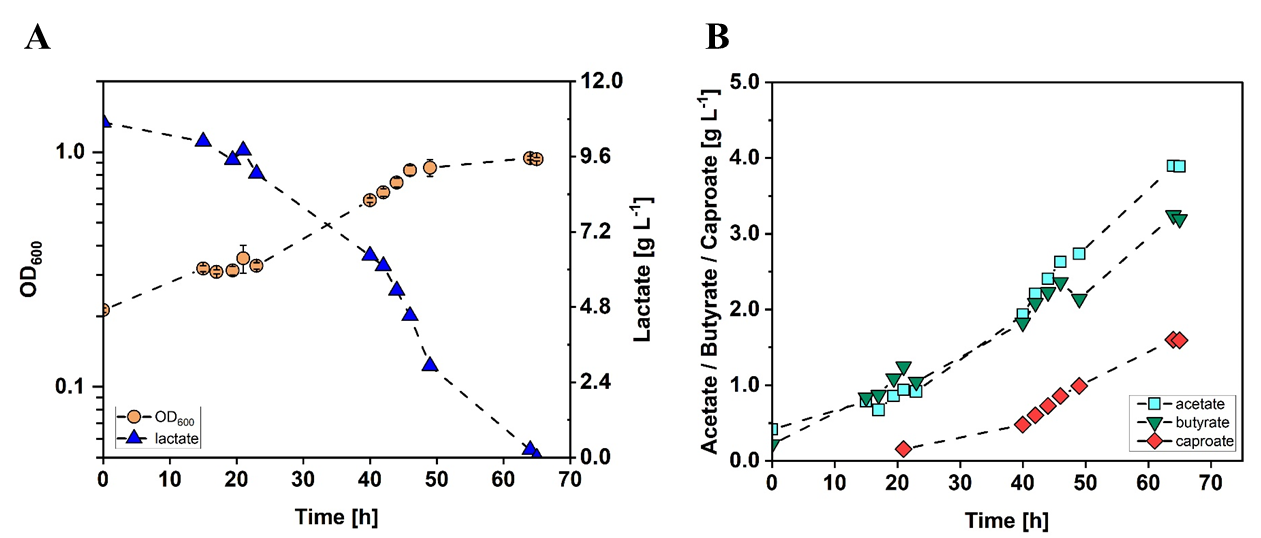


**Figure S2.** **Stirred-tank batch cultivation of *C. drakei* with lactate as substrate.**(A) Optical cell density (OD_600_, orange circles), lactate concentration measured in the medium (blue triangles); (B) acetate concentration (cyan squares), butyrate concentration (green triangles), caproate concentration measured in the medium (red diamonds). (T = 30 °C; pH = 7.0; P V^-1^ = 0.04 W L^-1^; F_CO2_ = 0.9 L h^-1^; V_0_ = 1.4 L).
